# Intraprocedural application of a peripheral blood flow monitoring system during endovascular treatment for femoropopliteal disease

**DOI:** 10.1016/j.jvscit.2023.101369

**Published:** 2023-11-20

**Authors:** John G. Winscott, Greg Stanley, Eric Scott

**Affiliations:** a Division of Interventional Cardiovascular Disease, University of Mississippi Medical Center, Jackson, MS; b Division of Vascular Surgery, Sanger Heart & Vascular Institute, Atrium Health, Charlotte, NC; c Division of Vascular Surgery, Iowa Methodist Medical Center, Des Moines, IA

**Keywords:** Angioplasty, Blood flow, Chronic limb threatening ischemia, Laser speckle imaging

## Abstract

Sensors that implement laser speckle image streaming provide real-time, noninvasive assessment of peripheral blood flow during endovascular revascularization. This single-center feasibility study evaluated a laser speckle-based peripheral blood flow monitoring system in 24 patients with peripheral arterial disease. System-quantified blood flow values showed improvement at the conclusion of the procedure in 20 of 24 patients (83.3%). Of the four patients without improved flow values, waveform morphology improved in three. Waveforms graded as moderate to severe peripheral arterial disease decreased from 71% before the procedure to 25% after the procedure, with improvement in 19 of 24 patients. In this limited population, laser speckle imaging could offer a highly sensitive method of detecting intraprocedural pedal blood flow changes.

Approximately 10% of people with peripheral arterial disease have chronic limb threatening ischemia (CLTI),^1^ the most severe form of the disease. CLTI is associated with higher amputation and mortality rates compared with less severe disease.^2^^,^^3^ Diagnostic technologies that provide quick and accurate peripheral vascular flow assessments are needed to support optimal management and outcomes.

The current standards of peripheral vascular assessment are the ankle brachial index and toe brachial index (TBI). However, the ankle brachial index and TBI are poor differentiators of the severity of arterial insufficiency.^4^, ^5^, ^6^, ^7^ Alternative noninvasive diagnostics include transcutaneous oxygen pressure monitoring, laser Doppler flowmetry, and photoplethysmography, each with their own limitations.^8^, ^9^, ^10^, ^11^, ^12^ Thus, an unmet need exists for a simple, noninvasive diagnostic tool for accurate real-time assessment of vascular flow.

Laser-speckle imaging uses laser light and imaging sensors to measure digital blood flow and assess vascular function. The FlowMet Peripheral Blood Flow Monitoring System (Medtronic) is a laser speckle imaging device in which a sensor is placed on a digit (typically the great toe) and laser light is transmitted from diodes through the tissue.^13^ A high-speed imaging sensor provides continuous blood flow measurements, displayed as changes in a speckle pattern, which is quantified using a calibrated numeric scale into a flow value and a waveform. The waveform reflects real-time changes in tissue blood flow during the cardiac cycle and enables characterization of blood flow as normal or abnormal, making it particularly amenable to intraprocedural monitoring.^14^ The waveform differs from a pulse volume recording tracing because it is derived from a direct blood flow measurement, rather than a blood pressure tracing. A prior feasibility study evaluated the laser speckle system in 90 patients and observed that the waveform morphology and amplitude were degraded in the setting of worsening arterial insufficiency and correlated with the TBI.^15^

## Methods

This single-center, prospective, all-comers pilot study evaluated the utility of the FlowMet Peripheral Blood Flow Monitoring System (Medtronic) in participants with peripheral arterial disease and planned endovascular revascularization.^15^ The study was conducted in accordance with the Declaration of Helsinki and approved by the institutional review board at the University of Mississippi Medical Center (Jackson, MS). All the patients provided written informed consent before enrollment.

Eligible patients were ≥18 years of age with infrainguinal disease and/or CLTI and a clinical diagnosis of peripheral vascular disease (Rutherford clinical class 2-6) requiring endovascular revascularization. Data were obtained from the medical records of diagnostic testing performed before, during, and immediately after revascularization.

The device was used to monitor tissue blood flow during the planned revascularization. On the procedure day and before generation of a sterile field in the catheterization laboratory, the reusable sensor was clipped onto a treatment limb toe, and the output was recorded using software on the connected tablet computer. The software recorded real-time flow values at a rate of 240 Hz for 30 seconds before the start of the procedure, throughout the revascularization procedure, and for 30 seconds after procedure completion.

**Fig 1:**
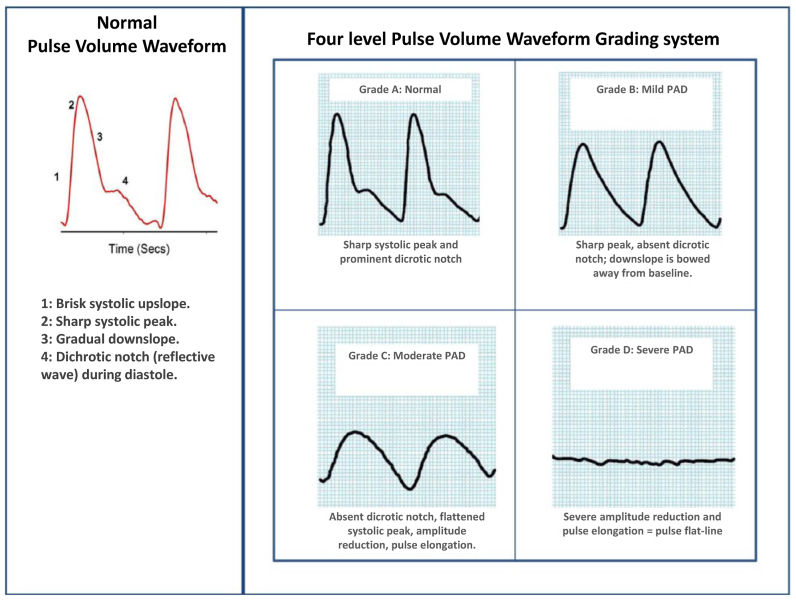
Four-level grading system for waveform characterization. *PAD*, Peripheral arterial disease. Defined by Rumwell and McPharlin^16^ in 1998, and reproduced, with permission, from Lewis et al,^17^ under the terms of a Creative Commons Attribution-NonCommercial 3.0 License.

The operator was unaware of the flow value and waveform results during the index procedure. Blood flow measurements were quantified using a unitless flow value. Waveforms were categorized using a four-level grading system^16^^,^^17^ (Fig 1) in a blinded independent review by three of us, with consensus determined by a two-thirds majority vote for each waveform.

No formal power or sample size computations were calculated. A sample size of 24 participants was considered sufficient to summarize the basic descriptive statistics to evaluate the device in this population. Basic descriptive statistics were computed for pre- and postprocedure timepoints and for the waveform change from before to after the procedure, which were categorized as improved, steady, or deteriorated. Continuous variables are summarized as the mean ± standard deviation and the median with the minimum and maximum values. Categorical variables are summarized as percentages and counts.

## Results

Thirty consecutive participants were screened and provided written informed consent. Twenty-four participants (30 limbs) were analyzed. Six participants were excluded because the data were diagnostic only and/or the target lesion could not be accessed or crossed.

### Baseline and procedural characteristics

The baseline demographic, clinical, and lesion characteristics are shown in the Table. The mean age of the patients was 66.9 years, and 75% were men. Most (91.7%) presented with Rutherford clinical category ≥4. Above-the-knee disease was treated in 45.8%, below-the-knee in 29.2%, and multilevel in 25.0%.

**Table:** Participant characteristics and outcomes

Characteristic	All participants (n = 24; n = 30 limbs)
Baseline	
Age, years	66.9 ± 10.0
Male sex	75.0 (18)
Body mass index, kg/m^2^	28.1 ± 6.4
Rutherford clinical class	
2	0.0 (0)
3	4.2 (1)
3-4	4.2 (1)
4	54.2 (13)
4-5	12.5 (3)
5	25.0 (6)
6	0.0 (0)
Level of disease treated	
Above the knee	45.8 (11)
Below the knee	29.2 (7)
Multilevel	25.0 (6)
Flow value	
Preprocedure	
Mean	5.2 ± 4.7
Median	3.6 (1.1, 20.2)
Postprocedure	
Mean	13.8 ± 12.9
Median	8.3 (1.3, 53.6)
Change	
Mean	8.6 ± 12.3
Median	4.4 (−11.2, 50.4)
Waveform	
Deteriorated	0.0 (0)
Steady	20.8 (5)
Improved	79.2 (19)

Data presented as mean ± standard deviation, percentages (counts), or median (minimum, maximum).

### Pre- and postprocedure results

**Fig 2:**
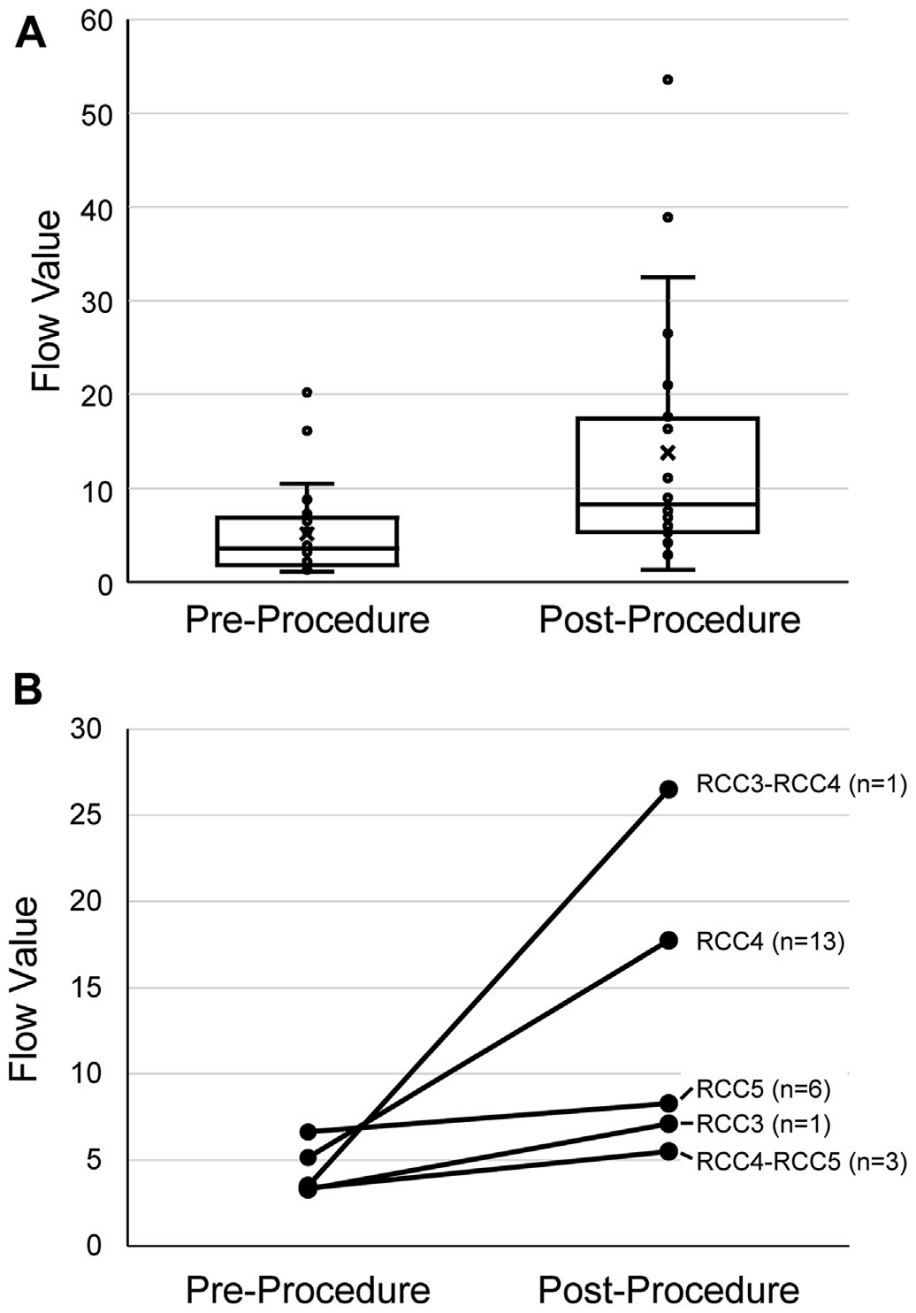
Flow values before and after the endovascular procedure. **A,** Flow values in all 24 patients. *Bars* from top to bottom are maximum, quartile 3, median, quartile 1, and minimum. The mean is shown with an *X*. **B,** Mean follow values before and after the procedure stratified by baseline Rutherford clinical class (*RCC*).

The mean flow value was 5.2 ± 4.7 before the procedure and 13.8 ± 12.9 after (Fig 2, *A*), increasing from before to after the procedure in 20 of 24 patients. Stratified by Rutherford class (Fig 2, *B*), the initial and ending flow values changed from 3.3 to 7.1 in the single Rutherford 3 participant, 3.5 to 26.5 in the single Rutherford 3 to 4 participant, an average of 5.1 to 17.7 in the Rutherford 4 participants (n = 13), 3.4 to 5.5 in the Rutherford 4 to 5 participants (n = 3), and 6.6 to 8.3 in the Rutherford 5 participants (n = 6). The average initial and ending flow values were 2.6 ± 1.7 and 13.5 ± 14.7 for those with above-the-knee disease (n = 11), 6.1 ± 4.8 and 12.4 ± 12.6 for those with below-the-knee disease (n = 7), and 8.7 ± 6.3 and 15.9 ± 11.6 for those with multilevel disease (n = 6).

**Fig 3:**
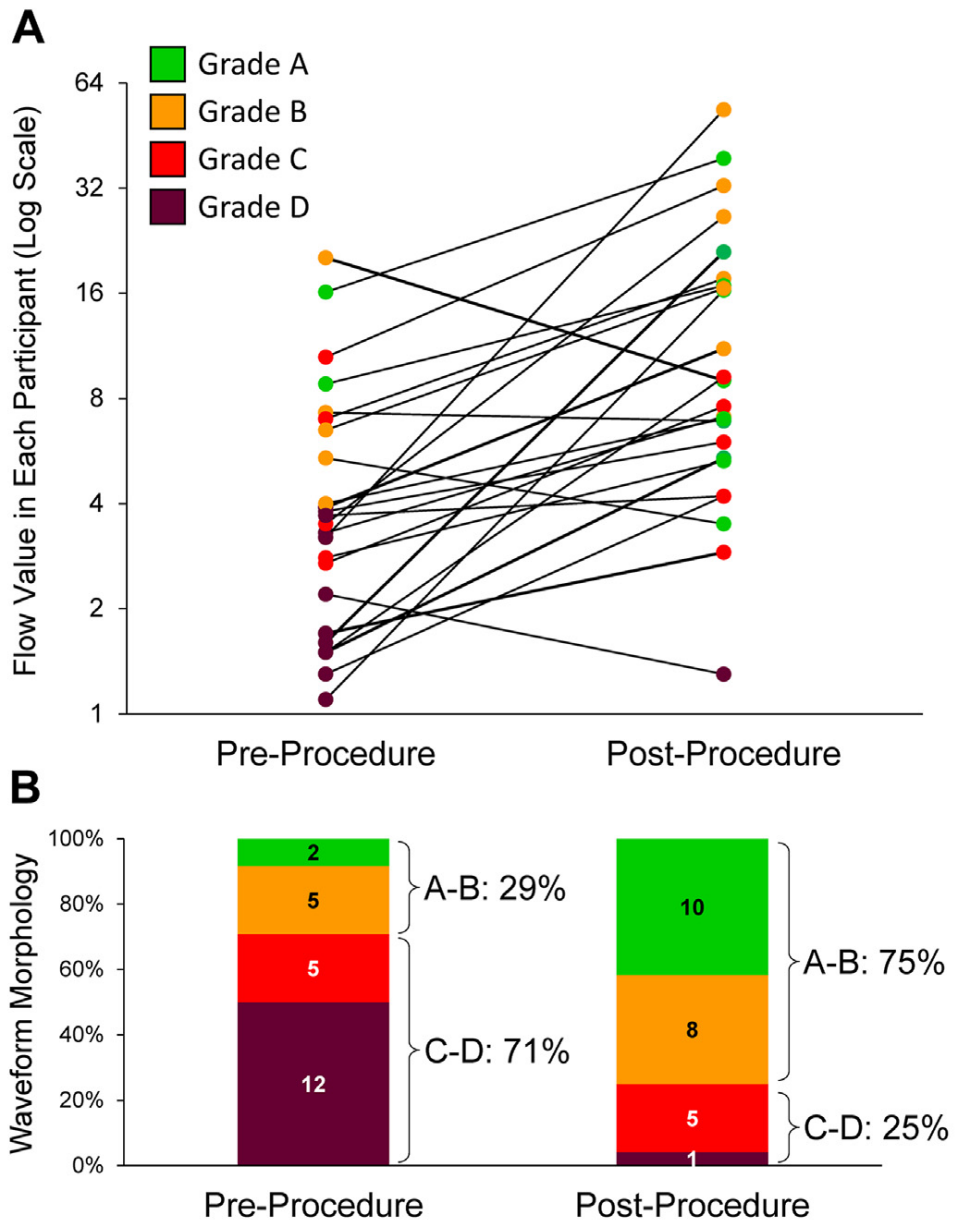
Flow values and morphology before and after the endovascular procedure. **A,** Individual participants shown using a log-2 scale before and after the revascularization procedure. **B,** Waveform morphologies before and after the endovascular procedure.

**Fig 4:**
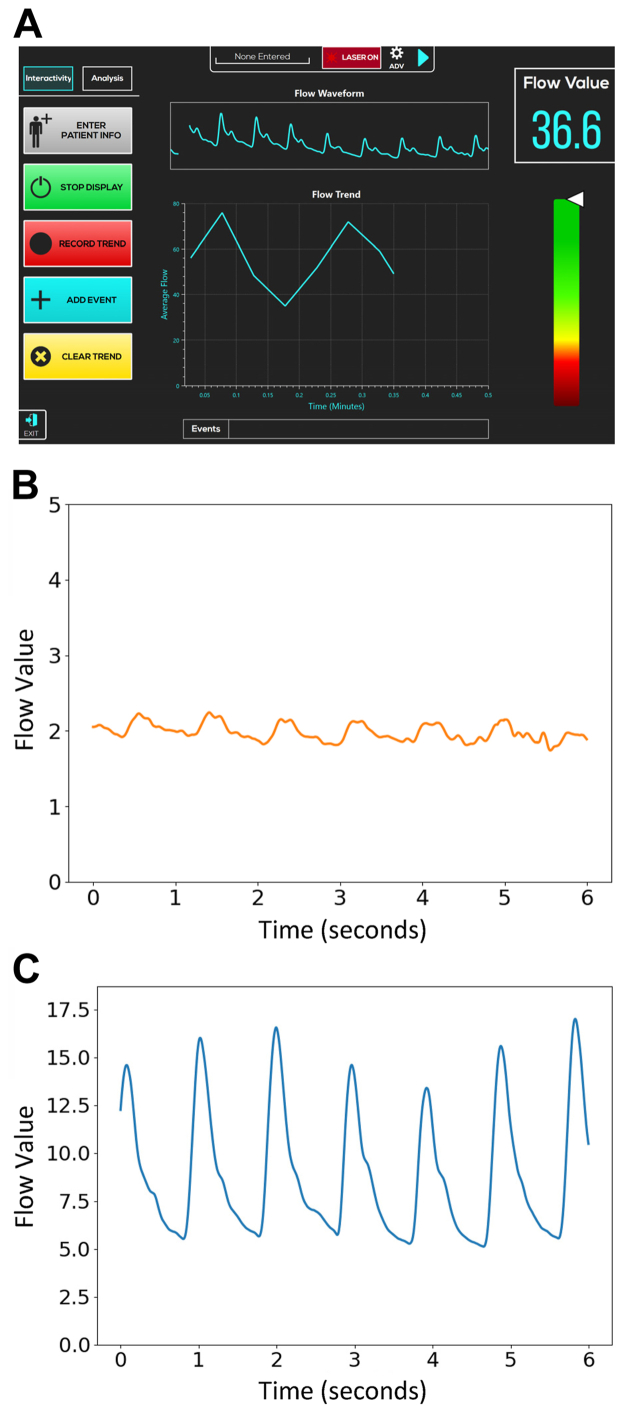
**A,** A generic example of the FlowMet Peripheral Blood Flow Monitoring System screen (Medtronic). **B,** An example waveform before the procedure. **C,** An example waveform after the procedure.

Using the categorizations shown in Fig 1, the waveform morphology readings improved from before to after the procedure in 19 patients (79.2%) and remained steady in 5 patients (20.8%; Table). The flow values and waveform morphology before and after revascularization are shown in Fig 3, *A*. For the four patients in whom the flow value decreased after the procedure, the waveform shape improved in three and remained the same in one. The percentage of waveforms graded as C to D decreased from 71% before the procedure to 25% after the procedure (Fig 3, *B*). Example waveforms are depicted in Fig 4.

## Discussion

The results from this study demonstrate the feasibility of a laser speckle blood flow monitoring system for use during peripheral endovascular procedures. The evaluated system generates a flow value and a waveform that closely resembles a pulse volume recording. The waveform depicts the loss of a dicrotic notch and progressive attenuation as the severity of arterial insufficiency increases. As a highly sensitive measure of flow, it demonstrated improvements in both flow value and waveform morphology after successful endovascular intervention. Although the average blood flow values were generally lower for patients with an increasing Rutherford class, deviations from this trend existed and were potentially due to the relatively small sample size. Further evaluation is required to explore how the flow value and waveform improvements seen in this study correlate with other established metrics of disease severity.

Real-time changes in pedal blood flow during peripheral revascularization procedures might allow physicians to monitor improvement, or the lack thereof, during the procedure and react accordingly. In particular, unexpected waveform depression during interventions provides immediate information regarding potential complications compromising flow that might not be otherwise detected until further angiography is performed. As shown previously, the waveform can help in relating system measurements to disease severity by supplementing blood flow measurements that might be altered by covariates such as edema or temperature.^15^ The ability to obtain this information in real time using a noninvasive system that does not require a large capital expense would be beneficial, in settings of both technical success and unanticipated complications.

## Conclusions

Although these results are promising, this small, single-center feasibility study needs to be replicated in larger, higher power studies that more thoroughly gauge performance and outcomes in claudication and CLTI settings. Furthermore, measurements outside the postprocedural observation period used in this study might further elucidate the effects of vasospasm or changes in vascular tone over time.

## Disclosures

J.G.W. declares consulting agreements with Cardiva, W.L. Gore & Associates, Medtronic, and Shockwave Medical. G.S. declares consulting agreements with Medtronic and Boston Scientific. E.S. declares consulting agreements with Medtronic and Boston Scientific.
